# Diabetes mellitus and risk of brain tumors: A meta-analysis

**DOI:** 10.3892/etm.2012.698

**Published:** 2012-09-06

**Authors:** JIAO JIAN TONG, HUANG TAO, OUYANG TAO HUI, CHEN JIAN

**Affiliations:** Department of Neurosurgery, Tongji Hospital, Tongji Medical College, Huazhong University of Science and Technology, Wuhan, Hubei 430030, P.R. China

**Keywords:** diabetes mellitus, brain tumor, epidemiology, meta-analysis

## Abstract

Accumulating evidence suggests that a history of diabetes may be involved in the occurrence of various types of cancer. However, the association of diabetes with the risk of brain tumors remains unclear. We identified relevant studies by performing a literature search of PubMed and EMBASE (through to 24 May 2012) and by searching the reference lists of pertinent articles. All data were extracted independently by two investigators using a standardized data abstraction tool. Summary relative risks (SRRs) with 95% confidence intervals (CIs) were calculated using a random-effects model. Inter-study heterogeneity was assessed using the Cochran’s Q and I^2^ statistical tests. A total of 13 studies were included in this meta-analysis, including the entire Danish population, 5,107,506 other participants and more than 2,206 cases of brain tumors. In the analysis of these 13 studies, we observed that diabetic individuals had a similar risk of brain tumors as non-diabetic individuals (SRR, 1.12; 95% CI, 0.89–1.42). There was significant evidence of heterogeneity among these studies (P<0.001; I^2^, 93.5%). Sub-group analysis revealed that diabetic females had a 24.2% increased risk of brain tumors (SRR, 1.242; 95% CI, 1.026–1.502), which was not observed in diabetic males. No significant publication bias was found in this study. The findings of this meta-analysis indicate that diabetic individuals have a similar risk of brain tumors as non-diabetic individuals. However, a significant positive correlation between the risk of brain tumors and diabetes mellitus was revealed in females, but not in males.

## Introduction

Cancer of the brain and central nervous system results in an estimated 142,000 mortalities per year, worldwide (1). The prognosis is poor for brain cancer patients, with 5-year survival rates of less than one-third (2). There are also indications that the incidences of glioma and meningioma have increased over the past few decades (3). However, there are few well-established risk factors for glioma and meningioma among adults. Although exposure to ionizing radiation and rare inherited genetic conditions, such as neurofibromatosis (4), are known to increase risk, these risk factors only explain a small fraction of reported brain tumors (5).

Diabetes mellitus (DM) is a serious and growing health problem worldwide and is associated with severe acute and chronic complications that negatively influence the quality of life and survival of affected individuals (6). DM has been recognized as a significant risk factor in several types of cancer, including cancer of the breast, endometrium, pancreas and liver (7–10). One mechanism to explain the correlation between DM and the risk of cancer is based on the hypothesis that the effect of the insulin and insulin-like growth factors (IGFs) axis triggers intracellular signaling cascades with mitogenic and antiapoptotic effects (11,12). Additionally, studies have found that DM patients have greater oxidative damage to their DNA as measured by the concentration of 8-hydroxy deoxyguanosine in mononuclear cells (13).

Due to inconsistent reports on the correlation between diabetes and brain tumor risk (14–26), the purpose of this study was to summarize all available evidence from published studies and to estimate the risk of brain tumors in patients with diabetes following the meta-analysis of the published studies. Available data were also analyzed according to the various study characteristics.

## Materials and methods

### Study identification

The electronic databases of PubMed and Embase were searched (up to May 24, 2012) using the following search terms: ‘diabetes’, ‘diabetes mellitus’, ‘DM’, ‘brain’, ‘CNS’, ‘Central Nervous System’, ‘cancer’, ‘neoplasm’, ‘tumor’, ‘incidence’, ‘risk’, ‘occurrence’, ‘mortality’ and combinations of these terms. All indexed studies were retrieved and we also reviewed the reference lists of the identified publications to discover additional pertinent studies. No language restrictions were imposed. The literature search was carried out independently by two investigators.

### Inclusion and exclusion criteria

The selection criteria were that the study: i) was published as an original article; ii) had DM as the exposure of interest; iii) had brain tumor incidence or mortality as the outcome of interest; iv) provided relative risk (RR), odds ratio (OR), hazard ratio (HR) or standardized incidence/mortality rate (SIR/SMR) with the corresponding 95% confidence intervals (CIs), or presented original data from which to calculate them; and v) at least took age as a confounding factor into consideration in the calculation of RR and corresponding 95% CIs. When there was overlap in the study populations between published papers, only the most recent or complete study was included.

### Data extraction

The following data from each included study were extracted using a standardized data-collection protocol: the first author’s name, country of origin, publication year, numbers of cases and subjects, sample size, definition of the study population, ascertainment of exposure and outcome, type of DM, participant characteristics (gender composition), duration of follow-up and variables adjusted for in the analysis. When several risk estimates were presented, we used those adjusted for the largest number of potential confounding factors. Data abstraction was performed independently by two investigators and then cross-checked.

### Statistical analysis

A meta-analysis of brain tumor risk was conducted. RRs were used as effect estimates. However, some studies reported using OR, HR or SIR estimates. Due to the rare occurrence of brain tumors, we assumed that all these measures would yield similar effect estimates and they were considered equally in the overall effect estimate. Summary RR estimates and the corresponding 95% CIs were calculated for all studies combined and by subgroups using the methods of DerSimonian and Laird with the assumptions of a random-effects model that considered intra- and inter-study variation (27). If studies reported RRs for each gender or various types of DM, we calculated a pooled RR and its corresponding 95% CI to determine the overall effect. Statistical heterogeneity between studies was evaluated using Cochran’s Q test and the I^2^ statistic (28). For the Q statistic, P<0.10 was considered to indicate statistically significant heterogeneity, and a value of I^2^>50% was also considered to indicate significant heterogeneity (29). Potential sources of heterogeneity were explored by meta-regression analysis. Funnel plots and Begg’s test were used to assess the potential publication bias (30). Statistical analyses were carried out with STATA version 11.0 (StataCorp, College Station, TX, USA). All tests were two-sided. P<0.05 was considered to indicate statistically significant differences.

## Results

### Search results and study characteristics

A total of 13 studies, including the entire Danish population, 5,107,506 other participants and over 2,206 cases of brain tumors, were found to match our inclusion criteria. Of these 13 studies, 4 were conducted in the Asia-Pacific region, 2 in the United States and 7 in Europe. Characteristics of the studies included in the meta-analysis are shown in Table I.

### Quantitative data synthesis

**Figure 1:**
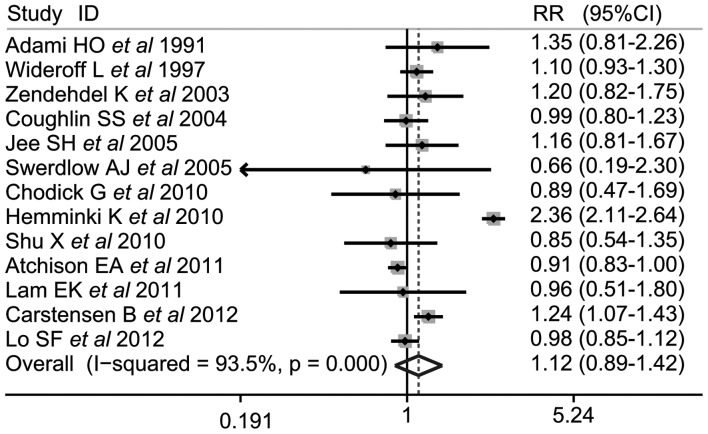
Forest plots of DM association with risk of brain tumors. Squares are study-specific relative risk. Diamonds are summary relative risks (SRRs). Horizontal lines represent 95% confidence intervals (CIs).

The combined results based on all studies demonstrated that there was a there was a similar correlation between DM and brain tumor risk (SRR, 1.12; 95% CI, 0.89–1.42; Q, 185.57; P<0.001, I^2^, 93.5%; Fig. 1).

We then conducted subgroup meta-analyses by gender, geographical region, types of DM and level of adjustments. A statistically significant positive correlation was detected between DM and brain tumor risk in females (SRR, 1.242; 95% CI, 1.026–1.502) but not in males (SRR, 1.024; 95% CI, 0.938–1.119), and there was clear heterogeneity in the analysis of female subjects (P=0.035; I^2^, 55.8%). Furthermore, positive associations were observed between diabetes and brain tumor risk in the diabetes assessment by self-report and by blood glucose level groups (SRR, 1.136; 95% CI, 1.017–1.268). However, no differences were found in brain tumor risk with diabetes between strata in the geographic region, types of diabetes, the number of cases, population size, duration of follow-up and level of confounding factors (Table II).

Meta-regression analyses were conducted to investigate the sources of heterogeneity between studies according to the above subgroups, however, we did not find a significant source of heterogeneity. Subsequently, we investigated whether the source of heterogeneity was a single study using a meta-regression analysis and found that the study by Hemminki *et al* (21) explained 88.91% heterogeneity.

A sensitivity analysis was carried out by omitting one study at a time and calculating the pooled RRs for the remainder of studies. The study by Hemminki *et al* (21) appeared to have a strong influence on the meta-analysis estimate of effect. After this study was excluded, we found that the pooled SRR changed slightly (SRR, 1.038; 95% CI, 0.950–1.134). We did not observe notable changes when the other studies were omitted.

**Figure 2:**
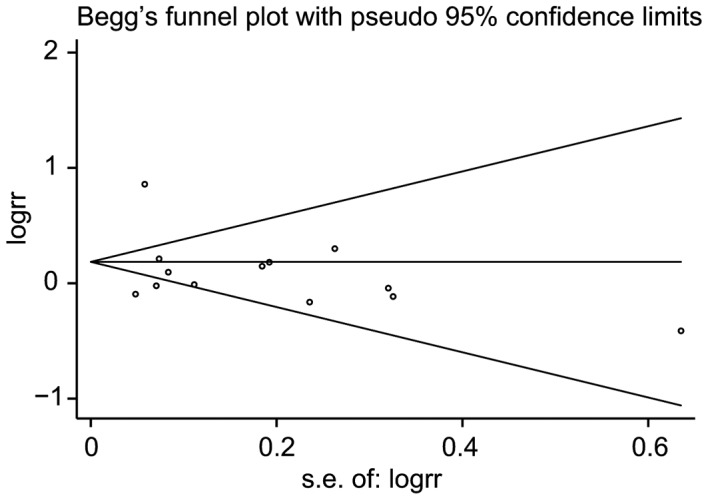
Begg’s funnel plot was used to detect publication bias in diabetes association with brain tumor risk

In the publication bias test the shape of the funnel plots appeared to be symmetrical for all studies investigating DM and the risk of brain tumors (Fig. 2). Begg’s test did not suggest any evidence of publication bias (P=0.669).

## Discussion

In this meta-analysis, we revealed that DM was associated with a 12% increased risk of brain tumors, however, this correlation was not statistically significant. However, a significant positive correlation between diabetes and brain tumor risk was observed in females, but not in males. This result was independent of the geographic region, type of diabetes, number of cases, population size, duration of follow-up and the level of confounding factors.

The null link between a history of diabetes and the risk of brain tumors is particularly notable since one of the hypothesized mechanisms for this association is via insulin resistance with secondary hyperinsulinemia. Hyperinsulinemia has been shown to increase the concentration of bio-available IGF-1 by reducing the concentration of IGF-binding proteins(31). The insulin and IGF axes are crucial in cell proliferation and apoptosis and thus may affect carcinogenesis(31,32). IGFs also exert an important role in the differentiation, proliferation and apoptosis of brain cells in early brain development and this may be a biologically feasible mechanism for an association between brain tumor risk and diabetes(33). However, it was demonstrated that although high concentrations of IGF-I are positively correlated with the risk of low-grade gliomas and acoustic neuromas, they are not correlated with the risk of high-grade gliomas and meningiomas(34).

Increased circulating insulin levels have a number of indirect effects, including decreasing the hepatic synthesis and blood levels of sex hormone-binding globulin, leading to increases in bio-available estrogen levels in males and females (12). Findings suggest that female sex hormones are protective against glioma (35). Since gliomas, including some of the most lethal types of cancer, account for over 80% of brain and central nervous system cancers (36), this may be a plausible mechanism to explain the null link between a history of diabetes and the risk of brain tumors when the large proportion of gliomas in brain tumor cases is taken into consideration.

In the subgroup analysis stratified by gender, our results showed that diabetes was associated with a significantly increased risk of brain tumors in females. Increased levels of insulin in blood circulation have been shown to induce an increase in the level of bio-available testosterone in females but not in males (12). Furthermore, obese males have lower levels of testosterone (37). There is evidence that testosterone stimulates cell growth and local production of IGF-I and IGF-I-R (38). This evidence may provide a plausible explanation for the positive correlation of brain tumor risk with diabetes in females, but not in diabetic males.

The strengths of the present study are as follows: i) our meta-analysis was based on 13 studies, the majority of which were prospective studies, thereby minimizing the possibility of recall or selection bias; ii) all included studies evaluated multiple potential confounding factors, some of which were considered to be risk factors for cancer, such as alcohol use, smoking and body mass index; iii) the varied populations of the studies expanded on prior observational studies by permitting additional subgroup evaluation (e.g., by gender, geographic region, type of DM and sources of population).

As with any meta-analysis of observational studies, there are several potential limitations to the results of this meta-analysis. Firstly, significant heterogeneity existed across studies, throwing some doubt on the reliability of the summary RR estimates. Although we found the main source of heterogeneity to be one study, we are unable to account for how this study differed from the others. Secondly, the majority of studies included in this meta-analysis did not distinguish between type 1 and type 2 DM. This non-differential misclassification may distort the magnitude of the association between DM and the risk of brain tumors. Thirdly, the history of DM may also reflect other factors associated with an unhealthy lifestyle, such as smoking, heavy alcohol consumption and obesity. Such unhealthy lifestyles have generally been associated with an increased risk of cancer. However, some authors did not adjust for those risk factors. Fourthly, the status of DM was self-reported in some studies. This may contribute to bias in the diabetes assessments. Additionally, the majority of studies did not consider the role of anti-diabetic drugs on the occurrence and mortality of brain tumors. This role may also contribute to bias. Finally, as in any meta-analysis, it is possible that an observed association is the result of publication bias, since small studies with null results tend not to be published. However, the results obtained from the funnel plot analysis and formal statistical tests did not provide evidence for such bias.

In summary, the results of this meta-analysis suggest a non-significant association between diabetes and the risk of brain tumors. However, compared with non-diabetic patients, diabetic females may have a slightly increased risk of brain tumors, whereas this was not the case in diabetic males. It should be noted that this meta-analysis does not provide firm evidence of any association between DM and brain tumor risk. Future studies are required to determine the role of a history of DM in brain tumor incidence or mortality.

## Figures and Tables

**Table I:** Characteristics of cohort studies of diabetes and brain tumor incidence and mortality.

First author (Refs.)	Source and starting-ending year	Study design	No. of participants	Diabetes assessment	Outcome ascertainment	Cases	Type of DM	FU (years)	Confounding factors
Adami HO (14)	Sweden 1965–1983	Cohort	51,008 (M/F)	Medical records	Cancer registry	66	No assessment	Mean 5.2	1,5
Wideroff L (15)	Denmark 1977–1989	Cohort	109,581 (M/F)	Discharge diagnosis	Cancer registry	159	No assessment	Mean 5.7	1,2,5,11
Zendehdel K (16)	Sweden 1965–1999	Cohort	29,187 (M/F)	Discharge diagnosis	Cancer registry	32	Type 1	Mean 14.4	1,2,5,11
Coughlin SS (17)	USA 1982–1998	Cohort	1,056,243 (M/F)	Self-report	Mortality registry	87	No assessment	Mean 14.7	1,4,7,8,9,10, 13,14,15,16
Jee SH (18)	Korea 1992–2002	Cohort	829,770 (M)	Self-report or blood glucose level	Cancer registry and records	NA	No assessment	Mean 10	1,3,8,9
Swerdlow AJ (19)	UK 1972–1993	Cohort	28,900 (M/F)	Discharge diagnosis	Cancer registry	16	Type 1 and 2	Mean 18	1,2,5,17
Chodick G (20)	Israel 2000–2010	Cohort	100,595 (M/F)	Self-report or blood glucose level	Cancer registry	12	No assessment	Mean 8	1,6,7,19,26
Hemminki K (21)	Sweden 1964–2007	Cohort	125,126 (M/F)	Medical records	Cancer registry	304	Type 2	Median 15	1,2,6,18,20,29
Shu X (22)	Sweden 1964–2006	Cohort	24,052 (M/F)	Discharge diagnosis	Cancer registry	20	Type 1	Mean 18.3	2,6,27,28
Atchison EA (23)	USA 1969–1996	Cohort	594,815 (M)	Discharge diagnosis	Cancer registry	527	No assessment	Mean 10.5	1,2,4,18,23
Lam EK (24)	Asia Pacific region NA	Cohort	367,361 (M/F)	Self report, diagnosis or blood glucose level	NA	168	No assessment	Median 4	1
Carstensen B (25)	Denmark 1995–2009	Cohort	The Danish population (M/F)	Medical records	Cancer registry	418	No assessment	NA	1,2,5,12,21,22
Lo SF (26)	Taiwan 1996–2009	Cohort	1,790,868 (M/F)	Medical records	Cancer registry	397	Type 2	Median 3.5	1,2,23,24,25

NA, not available; DM, diabetes mellitus; FU, follow up; M, male; F, female; Confounding factors: 1, age; 2, gender; 3, age2; 4, ethnicity; 5, calender year; 6, region; 7, body mass index; 8, smoking; 9, alcohol consumption; 10, red meat consumption; 11, excluding the first-year of follow-up; 12, excluding the first month-year of follow-up; 13, education; 14, consumption of citrus fruits and juices; 15, consumption of vegetables; 16, physical activity; 17, country-specific person-years at risk; 18, obesity; 19, cardiovascular diseases; 20, socioeconomic status; 21, duration of diabetes/insulin treatment; 22, date of birth; 23, chronic obstructive pulmonary disorder; 23, urbanization; 24, hypertension; 25, hyperlipidemia; 26, Supplemental Educational Services (SES) level; 27, age at first hospitalization; 28, period of diagnosis; 29, time period.

**Table II:** Subgroup analysis of relative risks for the association of diabetes with brain tumor risk.

			Heterogeneity		
Study	Studies	RR (95% CI)	Q	P-value	I^2^(%)
Total	13	1.121 (0.887–1.417)	185.57	<0.001	93.5
Gender					
Male	8	1.024 (0.938–1.119)	9.82	0.278	18.5
Female	6	**1.242 (1.026–1.502)**	13.58	0.035	55.8
Geographic region					
Asia Pacific	4	0.995 (0.879–1.417)	0.87	0.834	0
Europe	7	1.257 (0.888–1.779)	88.87	<0.001	93.2
North America	2	0.922 (0.846–1.005)	0.48	0.487	0
Types of diabetes					
Type 1	3	1.04 (0.803–1.348)	15.21	0.033	54
Type 2	3	1.177 (0.525–2.638)	97.13	<0.001	97.9
No assessment	8	1.061 (0.939–1.198)	15.21	0.033	54
Diabetes assessment					
Self-report or blood glucose level	6	**1.136 (1.017–1.268)**	4.41	0.492	0
Medical diagnosis or records	7	1.186 (0.831–1.693)	179.77	<0.001	96.7
Population size					
≥300,000	6	1.027 (0.909–1.161)	12.92	0.024	61.3
<300,000	7	1.192 (0.79–1.8)	77.94	<0.001	92.3
Cases among subjects					
≥150	6	1.199 (0.844–1.704)	177.8	<0.001	97.2
<150	6	1.023 (0.873–1.199)	3.17	0.674	0
Follow-up time (years)					
≥10	7	1.13 (0.733–1.741)	170.96	<0.001	96.5
<10	5	1.034 (0.935–1.143)	2.44	0.656	0
Level of considered confounding factors					
≥5	6	1.168 (0.809–1.685)	180.17	<0.001	97.2
<5	7	1.099 (0.969–1.245)	2.92	0.819	0

RR, relative risk; CI, confidence interval. Bold type indicates that the 95% Ci does not include 1.00.
